# Supplementary figures and images for: NMR resonance assignment and structure prediction of the C-terminal domain of the microtubule end-binding protein 3
Source: PLoS One. 2020 May 18;15(5):e0232338. doi: 10.1371/journal.pone.0232338 (PMC7233555; doi:10.1371/journal.pone.0232338)

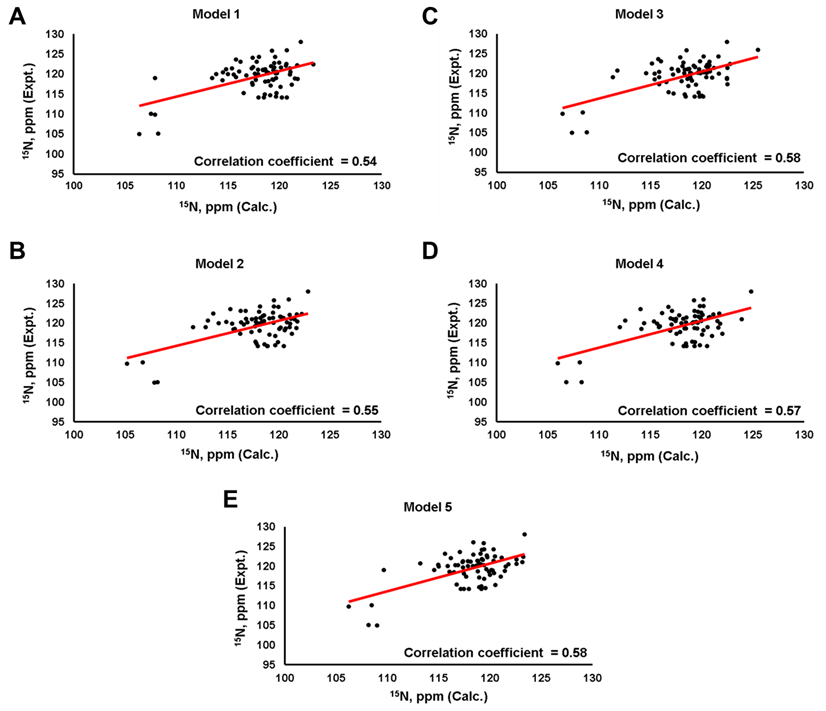

Supplement: S1 Fig — A-E) Comparisons of experimental 15N chemical shifts for the C-terminal domain of EB3 plotted along the Y-axis with the corresponding 15N chemical shifts calculated by SHIFTX 2.0 plotted along the X-axis; correlation coefficients are shown for each comparison. Models 3 and 5 of the C-terminal domain of EB3 exhibit the highest R2 correlation coefficients of 0.58. (TIF) [file pone.0232338.s001.tif]

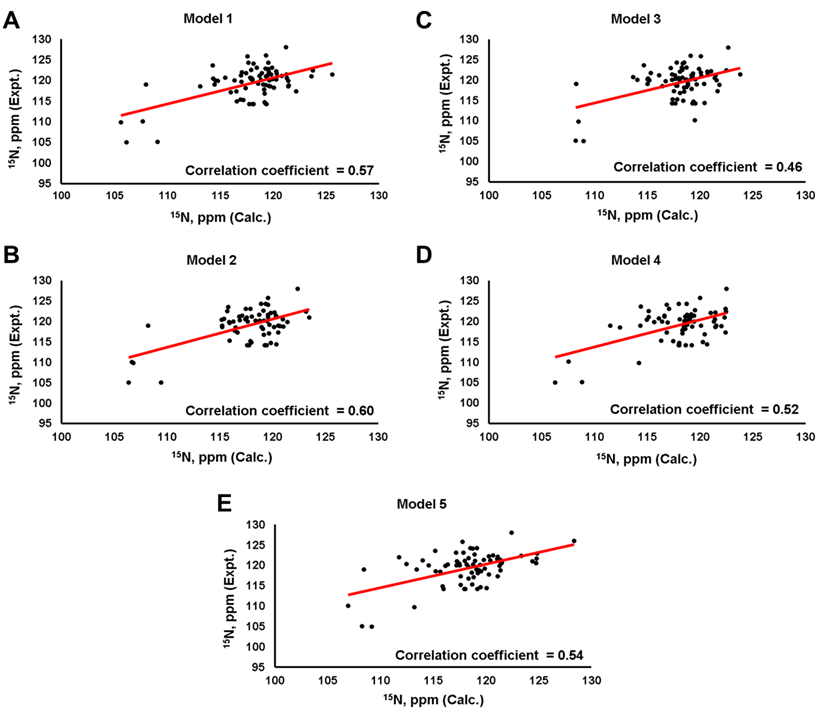

Supplement: S2 Fig — A-E) Comparisons of experimental 15N chemical shifts for the C-terminal domain of EB3 plotted along the Y-axis with the corresponding 15N chemical shifts calculated by SHIFTX 2.0 plotted along the X-axis; correlation coefficients are shown for each comparison. Model 2 of the C-terminal domain of EB3 exhibits the highest R2 correlation coefficient of 0.6. (TIF) [file pone.0232338.s002.tif]
