# Supplementary material for: NMR resonance assignment and structure prediction of the C-terminal domain of the microtubule end-binding protein 3
Source: PLoS One. 2020 May 18;15(5):e0232338. doi: 10.1371/journal.pone.0232338 (PMC7233555; doi:10.1371/journal.pone.0232338)
Supplement: Attachment — Submitted filename: Response to Reviewers.doc [file pone.0232338.s003.doc]

**Reviewer # 1 (Remarks to Authors)**

Major Comments:

1. There’s another BMRB file: ID ‘27311’ for NMR assignments of CTD of EB3 (residues 200-281) submitted by the same authors on 2017/11/13. Does the old submission contain incomplete assignments for the same protein fragment? How is the new submission (ID 50003, not available yet on BMRB) different from the old?

**We thank the reviewer for bringing this to our attention. The BMRB entry # 27311 contained incomplete assignments and has been removed from the database. The BMRB entry # 50003 will be available at the time of the manuscript publication.**

2. Figure 4 and page 6: There’s no BMRB file with ID 341191. Please fix to the correct ID: ‘34191’ in figure and text. Also, the R2 correlation coefficients are listed as 0.67 and 0.84 for IDs 18371 and 341191, respectively, in the text. However, these numbers are reversed in the figure. Please correct it.

**We have corrected these typos.**

Minor Comments

1. Abstract: Please include abbreviation ‘IP_3_R3’ for ‘inositol 1,4,5-triphosphate receptor 3, especially since the abbreviation is being directly used by the authors in the introduction afterwards.

**We have included the abbreviation in the abstract.**

2. Figure 1 shows poor resolution in this pdf file. Please check and provide a high-resolution image for the assignments.

**We have provided a new figure in the TIFF format with 300 psi resolution.**

3. Figure 2: Please correct the typographical error on the X-axis of all three panels to read ‘Residue Number’.

**We have corrected the x-axis labels in all panels.**

4. Figure 4: Please correct the typographical error on the X-axis of panels A, C, D, F, and G to read ‘15N, ppm (Calc)’.

**We have corrected the typo.**

**Reviewer # 1 (Remarks to Authors)**

Minor Comments

5. Figure 5, panel B: Please use uppercase ‘EB3’ instead of lowercase.

**Figure 5 is now numbered Figure 6 in the revised manuscript. We have also made sure the uppercase is used for EB3 everywhere in the manuscript.**

**Reviewer # 2 (Remarks to Authors)**

1) Sequence homology is very high between cEB1 and cEB3 – how different are the calculated structures generated using homology modeling solely based on sequence homology vs model structures reported in the manuscript. This is to show is there any contribution incorporating TALOS+ structural information in structure calculation?

**We have generated models of cEB3 based on the structure of cEB1 (PDB ID: 3GJO) alone, TALOS+ alone, or without homology and secondary structure restraints. Comparison of experimental and predicted ^15^N chemical shifts has shown that inclusion of cEB1 homology restraints alone (Fig. 5) or in combination with secondary structure restraints (Fig. 4) leads to the best agreement of the structural models with our NMR experimental data. However, the use of secondary structure restraints alone (S1 Fig.) or excluding secondary structure and cEB1 homology restraints (S2 Fig.) significantly reduced the agreement of structural models with the experimental data. Therefore, the best models could be built either using cEB1 homology restraints alone or in combination with TALOS+-derived secondary structure restraints.**

2) The authors observed concentration-dependent chemical shift change for several residues. Are these residues responsible for dimerization?

**We have clarified our statement that the residues exhibiting concentration-dependent chemical shift changes are found in helix 1, helix 2, and the loop region of cEB3. The corresponding regions in the structure of cEB1 (PDB ID: 3GJO) are involved in the homo-dimer interface.**
